# Direct and Indirect Induction of a Compensatory Phenotype that Alleviates the Costs of an Inducible Defense

**DOI:** 10.1371/journal.pone.0001084

**Published:** 2007-10-31

**Authors:** Tadashi Iwami, Osamu Kishida, Kinya Nishimura

**Affiliations:** Graduate School of Fisheries Sciences, Hokkaido University, Hakodate, Hokkaido, Japan; Oxford University, United Kingdom

## Abstract

Organisms often exhibit phenotypic plasticity in multiple traits in response to impending environmental change. Multiple traits phenotypic plasticity is complex syndrome brought on by causal relations in ecological and physiological context. Larvae of the salamander *Hynobius retardatus* exhibit inducible phenotypic plasticity of two traits, when at risk of predation by dragonfly larvae. One induced phenotype is an adaptive defense behaviour, i.e., stasis at the bottom of water column, directly triggered by the predation risk. Another one is a compensatory phenotype, i.e., enlarged external gills, for an unavoidable cost (hypoxia) associated with the induced defense. We identified two ways by which this compensatory phenotype could be induced. The compensatory phenotype is induced in response to not only the associated hypoxic conditions resulting from the induced defense but also the most primary but indirect cause, presence of the predator.

## Introduction

Inducible phenotypic plasticity represents the interplay between ecological factors and the organism's flexible developmental responses. Phenotypic responses are often thought to have an adaptive function to the primary deriving agent [1]–[4]. Defensive behavioural responses such as decreased activity and habitat changing are adopted by almost all mobile animals as part of their adaptive strategy against predation risk [5]. Although the defensive trait of prey organisms in response to predation risk provides a fitness advantage owing to avoidance of predation mortality, such traits are accompanied by unavoidable costs such as poor resource acquisition [6], [7]. Such unavoidable costs constitute a significant challenge to prey animals attempting to cope with the predatory environment, so the prey species might be expected to evolve some secondary means of dealing with these costs. Prey organisms might be able to both reduce their predation risk and lower their unavoidable cost by changing multiple traits simultaneously [8]. When a prey organism changes multiple traits in response to predation risk, the ecological significance of each trait and the functional relationships between them should thus be understood in terms of both predation risk reduction and compensation for the sequential unavoidable costs. In fact, several researchers have reported empirical evidence suggesting both predation risk and its associated costs induce organisms to change multiple traits [9]–[12].

Here we reported a remarkable induced morphology of a urodele species when at risk of predation by dragonfly larvae. We examined whether the inducible morphology is a secondary reaction that compensates for the cost incurred as a result of a primary indelible trait to the risk of predation. Further, we examined causal pathways via which inducing the secondary phenotype for the cost of the primary induced phenotype.

## Results

### Field observations

We found that *Hynobius retardatus* salamander larvae (a urodele species endemic in Hokkaido, Japan) in predator-abundant ponds have remarkably enlarged external gills compared to those in neighbouring ponds with few predators (see Methods). Although the enlarged gills seem to be a plastic response to the presence of predators in developmental period, they are not likely to have a defensive function, because enlarged gills, by increasing drag, might actually lower escape performance, and, moreover, might increase predation mortality by inviting the predator to attack the vulnerable head. Biologist has never known such enlargement of gills of urodele species when at risk of predation.

### Relationship between surfacing and dragonfly attacks

We found that *H. retardatus* salamander larvae reduced their surfacing frequency in the presence of larvae of the dragonfly *Aeshuna nigroflava*, the main predator of the salamander larvae in natural ponds (frequency without predators, 0.18±0.03 times/minute, mean±s.e.m., *n* = 14; frequency with predators, 0.02±0.01 times/minute, *n* = 14; *t*_26_ = 4.68, *P*<0.0001; statistical analyses were conducted on log-transformed data; see Methods).

In larval amphibians, a reduction of activity and suppression of surfacing in the presence of predators are considered to reduce their predation risk, because active prey are more likely to encounter and be detected by predators [13], [14]. To examine whether a decrease in surfacing frequency reduces predation mortality from dragonfly larvae, we investigated the relationship between surfacing of the salamander larvae and attacks by dragonfly larvae (see Methods). In the trials, nine of 13 salamander larvae that surfaced more than once during a ten-minute period induced attack behaviour of the dragonfly, whereas only one of 13 individuals that did not surface during the ten minutes induced attack behaviour (*χ* ^2^_1_ = 11.547, *P* = 0.0007). These results suggest that the salamander larvae reduce their predation risk by surfacing less frequently when dragonfly larvae are present. This adaptive behaviour, however, obviously subjects the salamander larvae to hypoxic conditions.

### Gill induction

In general, larval amphibians, including *H. retardatus,* take in oxygen not only from the water via their gills but also from the air by pulmonary respiration associated with a surfacing behaviour [15]. Reduction of surfacing might make it difficult for them to sustain their normal aerobic metabolism, and, in the long term, would affect growth and developmental rates [16]–[18]. Several species of salamander larvae plastically develop their external gills when exposed to hypoxic conditions [19], [20], and the enlarged surface area of this respiratory organ enhances oxygen absorption [21], [22].

**Figure 1:**
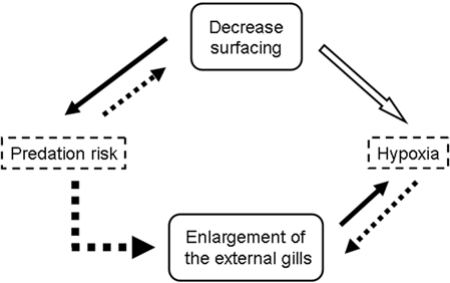
Conceptual schema of phenotypic plasticity in salamander larvae in the presence of dragonfly larvae. In this schema, “Predation risk” is the starting point and “Enlargement of the external gills” is the end point. Dotted arrows originate at possible signals, solid arrows show adaptive causal connections, and the hollow arrow points to an unavoidable cost. Thus, hypoxia is an unavoidable cost of the adaptive behavioural response “Decreased surfacing”. Enlargement of the external gills can be reached by two pathways, a multi-step pathway via the antipredator behaviour (decreased surfacing) and the consequent hypoxia, and a direct pathway via predator-produced cues.

We hypothesized that *H. retardatus* salamander larvae in the predator-abundant ponds might suffer from hypoxia for reasons related to the presence of predators and that the enlargement of their external gills might be an example of adaptive phenotypic plasticity to enhance their oxygen uptake under water for coping with hypoxic conditions, and that the direct cue for enlargement of the external gills might be hypoxic conditions. However, since the hypoxic conditions are strongly linked to an adaptive defensive behaviour, we also inferred that the plastic enlargement of the gills might be directly cued by signals from the predatory dragonfly larvae. Therefore, we hypothesized two possible causal pathways leading to enlargement of the external gills: a multi-step pathway, via the anti-predator behavioural change and the consequent deficiency of oxygen uptake (hypoxia); and a direct pathway, via predator-derived signals of predation risk (Figure 1).

**Figure 2:**
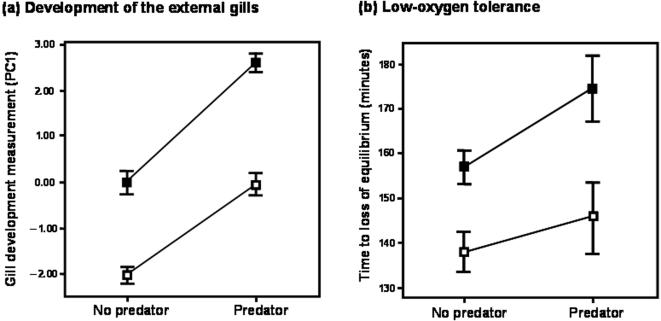
(a) Effects of predator presence and oxygen availability on the development of the external gills of salamander larvae. (b) Low-oxygen tolerance of salamander larvae reared under predator presence or absence, and high- or low-oxygen treatment. Open and solid squares indicate that rearing condition of larval salamander is the high-oxygen treatment and the low-oxygen treatment, respectively. Error bars indicate ±1 s.e.m.

**Figure 3:**
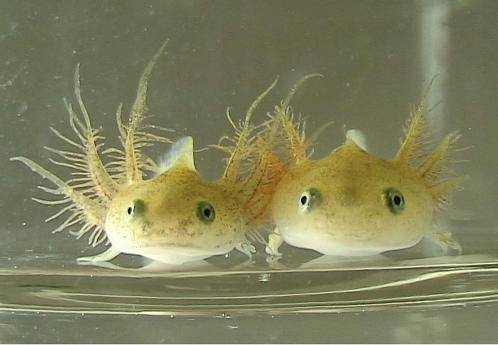
Representative salamander larvae reared under low-oxygen conditions in the gill induction experiment. The individual on the left was subjected to the predator treatment and the one on the right to the no-predator treatment.

We performed an experiment to determine whether the plastic response in external gill morphology was induced by hypoxia or predation risk, or both. We compared the responses in salamanders exposed to high and low levels of oxygen concentration and high and low levels of predation risk (see Methods). After rearing salamander larvae in each condition for 22 days, we measured five gill morphological variables to quantify development of the external gill. We then analyzed the gill morphological variables by a principle component (PC) analysis, and interpreted PC1 as an indicator of the development of the external gills (see Methods). The results of a two-way nested analysis of variance (ANOVA) of the value of PC1 indicated significant effects of both predation risk (*F*_(1,14.04)_ = 40.16, *P*<0.0001, Figure 2-(a)) and oxygen concentration (*F*_(1,14.04)_ = 41.98, *P*<0.0001, Figure 2-(a)), but no interaction effect between them (*F*_(1,14.04)_ = 0.79, *P* = 0.39, Figure 2-(a)). The salamander larvae subjected to a low oxygen concentration had larger external gills than those subjected to a high oxygen concentration, as did those exposed to high predation risk (Figure 3). This result showed that the salamanders enlarged their external gills via both causal pathways, the multi-step pathway, from the anti-predator behavioural change to hypoxia to gill enlargement, and the direct pathway, from predation risk to gill enlargement.

### Low-oxygen tolerance

We also investigated the low-oxygen tolerance of individual larval salamanders reared in each of the four treatment combinations (see Methods). We then subjected these individuals to acute hypoxic conditions, and recorded the time until they lost their equilibrium posture (an indication of approaching death). The results of a two-way nested ANOVA of hypoxia tolerance time (Figure 2-(b)) showed a pattern identical to that shown by the results of the gill induction experiment. Salamander larvae reared at a low oxygen concentration or under predation risk maintained their equilibrium posture longer than those reared at a high oxygen concentration or under no predation risk (*F* and *P* values of the predator environment and oxygen concentration and of their interaction effects were *F*_(1, 13.87)_ = 9.33, *P* = 0.009; *F*_(1, 13.87)_ = 28.88, *P*<0.0001; and *F*_(1, 13.87)_ = 1.77, *P* = 0.205, respectively, Figure 2-(b)).

## Discussion

*Hynobius retardatus* salamander larvae exhibited plasticity in both defensive behavioural and respiratory traits in the presence of predator *A. nigroflava* dragonfly larvae; that is, they surfaced less frequently (anti-predatory adaptive behavioural plasticity) and enlarged their external gills (adaptive morphological plasticity in response to hypoxia caused by the anti-predatory behaviour).

A remarkable finding of this study is that multiple pathways exist via which the secondary phenotype compensating for the unavoidable cost incurred as a result of the primary adaptive phenotypic plasticity can be induced. Many adaptive plastic responses have been studied in other organisms, and it is well known that organisms change traits in response to direct signals from the objects to which they need to adapt [2], [but see 10], [23]. In this study, however, the salamander larvae change their morphology in response not only to hypoxic conditions but also to predation risk, in relation to which the enlarged gills are not directly adaptive. Since the unavoidable cost of hypoxia is closely linked to the presence of a predator and the enlargement of the external gills compensates for that cost, the induction of gill enlargement by predator cues is unlikely to turn out to be a false decision. Furthermore, expression of the compensatory phenotype via the multi-step pathway might require considerable time, whereas responding directly to predator cues allows immediate compensation for the cost (hypoxia) incurred.

Furthermore, high tolerance for low oxygen in individuals reared under low-oxygen conditions and predation risk might possibly not result solely from the enlargement of the external gills. Other possible physiological plastic responses such as increases in heart rate and hematogenesis and many other cryptic mechanisms to improve oxygen uptake [24], [25] might be induced concurrently by these environmental signals.

Compensatory phenotypic plasticity cannot completely eliminate unavoidable costs associated with the primary defensive response, and the compensatory response itself may be costly to produce or maintain (although that cost should be off set by the resulting fitness benefit). For these reasons, the two traits (i.e., the defensive behaviour and enlarged external gills) are inducible by predation risk rather than maintained regardless of predation risk. In this way, prey organisms can maximize their fitness by exhibiting the defensive behaviour and compensating for the consequent unavoidable cost when they are exposed to predation risk, whereas they can save the costs incurred as a result of these induced responses when they are in a safe environment [26].

An adaptive phenotypic response can impose a new challenge that might demand a new adaptive phenotypic change. Thus, the functional relationship between multiple induced phenotypes may be more sophisticated than we previously thought. Functional interaction among multiple inducible traits may turn out to be the prevalent design in organisms inhabiting variable selective environments.

## Materials and Methods

### Gill measurements

**Figure 4:**
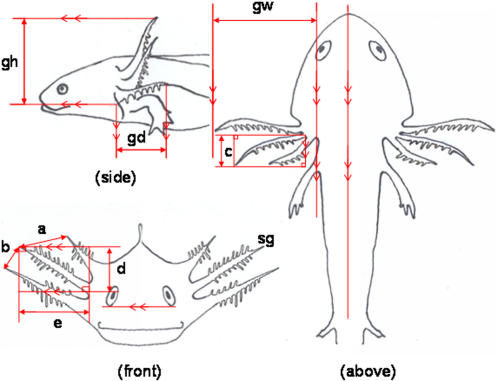
Morphmetric measurements of gill rachises. Larval salamander gill measurements. sg, second gill; gh, gill height; gd, gill depth; gw, gill width; a+b, distance between the three gill rachises; 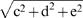, second gill length.

We quantified the development of external gill morphology of *H. retardatus* salamander larvae in the field and in laboratory experiments. We measured five variables: gill height and gill depth (side view), gill width (top view), distance between the three gill rachises (front view) and the length of the second gill rachis (top and front views) (illustrated in Figure. 4). These variables were measured on images photographed with a digital video camera (Sony Handycam DCR-PC1000) from three orthogonal directions. The second gill length was calculated according to the Pythagorean Theorem. We considered these morphological variables to represent gill size and gill spatial extension, and all of the variables together to indicate the development of the external gills. To reduce the number of dimensions needed to describe the variation in the development of the external gills, we analyzed the data of the five morphometric measurements by principal component analysis (PCA), using the correlation coefficient matrix. The major axis (PC1) in PCA of salamander external gill morphology accounted for 85.1% (field observations at Higashimata; *n* = 22), 67.8% (field observations at Nishimata; *n* = 50) and 75.8% (in the gill induction experiment; *n* = 88) of the total variance of all gill morphological variables. Since the sign of all elements of the eigenvector for PC1 was positive in each analysis (field, Higashimata and Nishimata; laboratory, the gill induction experiment), we interpreted PC1 to reflect the development of the external gills.

### Field observations

We compared the development of external gills of salamander larvae between predator-abundant ponds and ponds with few predators. In early July 2005, we surveyed two sites (Higashimata and Nishimata) alongside a forest road that follows branches of the Moheji River, Hokkaido, Japan. At each site, there were two neighbouring ponds, a predator-abundant pond and one with few predators). We estimated densities of invertebrate predators and salamander larvae by using sampling the populations by net according to a standard procedure [27]. The salamander larvae were sampled when most of the population was in the middle developmental period before metamorphosis. In that developmental period, the concentration of dissolved oxygen in the water fell within the following ranges: at Higashimata, predator-abundant pond, 2.55–4.76 mg/ℓ; low predator density pond, 3.41–3.67 mg/ℓ; and at Nishimata, predator-abundant pond, 4.11–5.59 mg/ℓ; low predator density pond, 4.36–5.60 mg/ℓ. At each site, the predator-abundant pond contained a single invertebrate predatory species: at Higashimata the predator was larvae of the dragonfly *Aeshuna nigroflava* (14.9/m^2^; individuals/m^2^), and at Nishimata, it was larvae of the diving beetle *Dytiscus marginalis* (2.4/m^2^). The estimated density of the salamander larvae in the predator-abundant and the low predator density ponds was 41.3/m^2^ and 83.3/m^2^, respectively, at Higashimata, and 126.9/m^2^ and 150.0/m^2^, respectively, at Nishimata. We collected 10–29 salamander larvae (stage 48–62 [28]) from each pond, measured their body mass and external gill morphometrics, and quantified the magnitude of their gill development. We compared the mass-adjusted PC1 of individuals from the predator-abundant pond and low predator density pond at each site to determine if development of the external gills of the salamander larvae differed between them. At both sites, the salamander larvae had larger gills in the predator-abundant pond than in the low predator density pond. At Higashimata, mass-adjusted PC1 values for individuals from the predator-abundant and the low predator density ponds were 1.71±0.19; mean±s.e.m. (*n* = 10) and −1.37±0.11 (*n* = 12), respectively (*t*_20_ = −14.45, *P*<0.0001), and at Nishimata, they were 0.74±0.29 (*n* = 21) and −0.72±0.18 (*n* = 29), respectively (*t*_48_ = −4.47, *P*<0.0001).

### Collection and maintenance of experimental animals

Egg clusters of the salamander *H. retardatus* and the late instar dragonfly *A. nigroflava* larvae used in the laboratory experiments were collected from several natural ponds on Oshima Peninsula in Hokkaido, Japan. Collected salamander eggs were kept in 4.3-ℓ (surface area, 29×16.5 cm; 9 cm high) tanks, and dragonfly larvae in 0.5-ℓ (surface area, 12×8 cm; 5 cm high) tanks. Salamander hatchlings were fed chironomid larvae every day ad libitum. Dragonfly larvae were fed salamander larvae every three days ad libitum. Rearing water of the animals was changed every three days. The animals were maintained and the experiments were conducted in a laboratory at 16°C under a natural day/night regime (14 h/10 h).

### Relationship between surfacing behaviour and dragonfly attacks

We examined surfacing frequency of the salamander larvae in the presence of predation risk by dragonfly larvae. Twenty-day-old salamander larvae (stage 55–62; 0.23±0.01 g; mean mass±s.d., *n* = 28) were individually assigned to experimental tanks (surface area, 44×32.5 cm; 16 cm high) filled with 20 ℓ of tap water. We conducted two treatments (i.e., predator and no-predator treatments), each with 14 replicates. In each predator treatment tank, we placed a caged dragonfly larva and added 100 mℓ of dragonfly-conditioned water. In each no-predator treatment tank, we placed an empty cage and added 100 mℓ of tap water. Salamander larvae were acclimated in each tank for an hour, and then we recorded surfacing frequency during a 30-minute period. We compared the surfacing frequency (times/minute) of the salamander larvae between the treatments by *t*-test. The data on surfacing frequency were log-transformed to meet the assumptions of the parametric test.

To determine whether decreased surfacing frequency of the salamander larvae might reduce predation mortality caused by attacking dragonfly larvae, the relationship between salamander surfacing behaviours and dragonfly attacks was examined. Two late instar dragonfly larvae were assigned to experimental tanks (surface area, 44×32.5 cm; 16 cm high) filled with 15 ℓ of tap water and 100 g of sand. We conducted the trials after the dragonfly larvae had acclimated to the experimental setting for ten days. First, we divided the tanks in half with an opaque plastic board, and the dragonflies were placed on one side. On the other side, we placed a small clear plastic box (surface area, 21.5×12.5 cm; 13 cm high) filled with environmental water (3 ℓ of tap water and 100 mℓ of dragonfly-conditioned water). Then, we placed one salamander larva (20 days old, stage 55–62, 0.48±0.092 g; mean mass±s.d., *n* = 26) into the small box. After the salamander larva stopped moving, we quietly removed the opaque plastic board, and observed the surfacing behaviour of the salamander larva and the attack behaviour of the dragonfly larvae. Here, we defined rushing behaviour or labium elongation of the dragonfly larvae toward the salamander larva within the box as attack behaviour. These trials were replicated in 26 tanks.

### Gill induction

We performed an experiment to determine if the plastic response in external gill morphology was induced by hypoxic conditions or by predator cues indicative of predation risk or both. We conducted a complete factorial experiment, in which two levels of oxygen concentration were crossed with two levels of predation risk. A tank (surface area, 29×16.5 cm; 9 cm high) containing 3.8 ℓ of water and six cylindrical column-shaped mesh cages was used as the experimental unit. Five two-day-old salamander larvae (snout–vent length = 13.14±0.54 mm; mean±s.d., *n* = 20) were individually assigned to five of the mesh cages in each tank. In each tank of the predator treatment, a late instar dragonfly larva was assigned to the remaining cage (the sixth cage was left empty in the no-predator treatment tanks). For the high-oxygen treatment, we maintained a high oxygen concentration (5.99–6.32 mg/ℓ) by continuously bubbling atmospheric air through the tanks, and for the low-oxygen treatment, we maintained a low oxygen concentration (3.00–3.37 mg/ℓ) without bubbling. We performed five replicates of the four treatment combinations. We fed all salamanders the same quantity of chironomid larvae every day. Dragonfly larvae were replaced with individuals from the stock tank and fed 100 mg of salamander larvae in the experimental tanks every three days. Since in this experiment, our focus was on the external gill plasticity directly cued by oxygen concentration or predator cues, plastic responses to differences in individual oxygen demand caused by differences in surfacing frequency between treatments were not of interest. Therefore, we prevented all salamander larvae from surfacing by covering the column-shaped cages with clear plastic boards under the water surface. After rearing the salamanders for 22 days (to stages 55–62), we measured the five gill morphological variables and body mass to quantify the development of external gill morphology (see section on gill measurements). Preliminary analysis by nested ANOVA of the gill development indicator (PC1), in which a predator effect and an oxygen effect were the main fixed factors, tank effect was a random factor, and body mass was a covariate factor, and the interactions among factors, showed that the covariate effect and its interaction effects were not significant. Therefore, we pooled the covariate effect and its interaction effects and their degrees of freedom into the error term and conducted the statistical analysis by nested ANOVA. In these analyses, we excluded data from two tanks (one tank from the low-oxygen and predator treatment, and the other from the low-oxygen and no-predator treatment), because of abnormal development of larvae. In addition, we also excluded two salamander larvae, one from the low-oxygen, no-predator treatment and one from the high-oxygen, no-predator treatment, because they were injured accidentally.

### Low-oxygen tolerance

We conducted an experiment to investigate the low-oxygen tolerance of individuals reared in each of the four treatment combinations in the gill induction experiment. After we had measured the gill morphological variables in the gill induction experiment, we used some of the measured individuals in this experiment. We submerged 40 cylindrical column-shaped net cages in a 180-ℓ aquarium (surface area, 90×45 cm; 45 cm high) filled with 90 ℓ of tap water. We randomly collected salamander larvae from each treatment (10 individuals×4 treatments) and placed them individually in the 40 cages. We prevented them from surfacing by covering the column-shaped cages with clear plastic boards. After all individuals were in place, we gradually decreased the amount of dissolved oxygen in the water by continuously bubbling N_2_ gas into the tank. We circulated the water in the tank by an internal pump to keep the dissolved oxygen concentration uniform at about 0.4 mg/ℓ throughout the tank during the experiment. We then recorded the time until the salamander larvae lost their equilibrium posture as a measure of low-oxygen tolerance (loss of equilibrium precedes death; individuals that lost equilibrium were removed from the tank and allowed to recover in tap water saturated with oxygen). We conducted a statistical analysis by nested ANOVA of the time to loss of equilibrium, which involved the factors predation risk, oxygen concentration, tank, and their interaction.
